# Descriptive study of how proportioning marks determines the performance of nursing students in a pharmacology course

**DOI:** 10.1186/s12912-020-00506-x

**Published:** 2020-11-30

**Authors:** Sheila Anne Doggrell

**Affiliations:** Faculty of Health, Queensland University of Technology, Brisbane, QLD 4002 Australia

**Keywords:** Assignment, Examinations, Marks, Nursing students, Ongoing assessment, Pharmacology, Tutorials

## Abstract

**Background:**

In programs with higher proportions of marks allocated to ongoing assessment, the students have higher overall marks than those with a lower proportion allocated to assessment. Little or no attention has been made to how the allocation affects the academic success of students in individual courses. The purpose of this study was to determine how the allocation of marks to examinations, tutorials and an assignment affects the performance of nursing students in a pharmacology course.

**Methods:**

For students who passed a pharmacology course (i) the marks for examinations and ongoing assessment (tutorials and/or an assignment) were compared, and (ii) regression line and correlation analysis was undertaken to determine any association between these marks. In addition, for completing students, modelling was undertaken to determine the effects of changing the allocation of marks on passing and failing rates.

**Results:**

Nursing students who passed a pharmacology course obtained significantly lower marks in examinations than ongoing assessment, and for the ongoing assessment, lower marks in the assignment than tutorials. Regression line analysis showed that the marks in ongoing assessment (tutorials and/or the assignment) versus examination marks were a poor line-fit. The correlation coefficients between ongoing assessment and examinations were weak to moderate. A high percentage of students passed the course (> 90%) and, modelling for completing students, showed that decreasing the marks for examination would have led to slightly more students passing the pharmacology course with higher grades. In contrast, increasing the marks for examination would have dramatically decreased the number of students passing the course, and their grades.

**Conclusions:**

The allocation of marks can have a major effect on student performance. As ongoing assessment is only a weak or moderate indicator of performance in examination this has implications for students who rely on passing examinations for their advancement. For instance, nursing students in some countries (e.g. USA) are required to pass examinations prior to registration, whereas in others (e.g. Australia) they are not. Consideration needs to be given as to whether it is appropriate for nursing students who fail examinations to pass courses/programs.

## Background

Historically, examinations, which students had no prior access to, were the most common way to determine academic performance for students. However, over the last 40 years, ongoing assessment (coursework) has been introduced into many degrees [1], so that most courses have become a mixture of examination and ongoing assessment. Presently, examinations are often used to test the assimilation of knowledge and ensure that the students complete the work themselves [1]. However, due to time pressures, examinations do not allow academic excellence whereas ongoing assessment is used to teach as well as test [1].

There are no rules about the proportional allocation of marks for ongoing assessment and examination, and the allocation is often made on a seemingly arbitrary basis and not justified. For instance, in pharmacology courses for nurses in Australia, the proportion of marks for examinations ranges from 40 to 80% [2–6]. The consequences of the proportional allocation of marks in courses are often not considered.

Assessment can either be summative, which evaluates student learning at the end of the component or course, or formative, which monitors students learning to provide ongoing feedback. Whereas examinations are clearly summative, ongoing assessment can be either summative or formative. One of the reasons for this is that ongoing assessment takes many forms including weekly quizzes, homework, tutorials, laboratory work, oral or poster presentations, and assignments/research projects [7]. Some of these ongoing assessment types are examples of formative activities e.g. weekly quizzes and homework, whereas others are summative e.g. final presentations and final reports [8].

There is evidence that the marks for ongoing assessment are higher than for examinations, and this has various consequences. Across UK universities, in the programs with higher proportions of ongoing assessment, students had higher overall marks, and consequently better degrees, than those with a lower proportion of ongoing assessment [9–11]. This also applies to students in biology/molecular sciences having higher marks in courses with 100% assessment, compared to courses with mixed assessment [12].

There have been few studies of the association between marks in ongoing assessments and examination in single programs or courses. Studies have shown that the marks for ongoing assessment were higher than examination marks in a pharmacy program [13] and in a bioscience course [14]. However, it is not known whether this applies to all kinds of ongoing assessment versus examinations, and to all students and courses/programs.

The relationship between marked examinations and formative unmarked ongoing assessment has been considered in meta-analysis. This meta-analysis was of the effect of active learning interventions on examination outcomes in the STEM disciplines and showed that the interventions improved examination marks by 6%, and reduced failure rates compared to traditional lecturing [15]. The interventions were unmarked formative activities such as worksheets or tutorials completed during class [15]. Notably, formative activity that was marked, such as worksheets/homework completed prior to tutorials/workshops, were not included in this meta-analysis.

There have been few studies of the relationship between marked formative or summative ongoing assessment and marks in examinations, and these have had varying outcomes. In a pharmacy program, there was only a weak correlation between the marks for ongoing assessment and examinations and no correlation between the marks for a practical write-up and an aligned examination question [13]. In contrast, marks for home assignments were a strong predictor of examination performance in courses in calculus and macroeconomics [16], and education [17]. Marks for home assignments in statistics were shown to predict examination performance in one study [18], but not another [16]. Other studies have shown that marked tutorial-based assessments have a significant positive association with examination performance in finance [19] and law [20] courses. Marked online quizzes were also associated with better performance in examinations for education students [17].

Being able to perform well in examinations is especially important for nursing students as it may determine whether they can practice clinically. There are differences in how nursing registration is achieved between countries. In the USA and Canada, it is the marks in national examinations taken after completing their studies, which determines whether the students can practice. In contrast, Australia, the UK, the Republic of Ireland, and New Zealand are among the countries not requiring national examination prior to registration for nursing students but relying on graduation from courses with examinations and ongoing assessment.

### Research questions and hypothesise

(i) Do nursing students have higher marks in ongoing assessment than examinations? The hypothesis was that they would.
(ii) Do marks in ongoing assessment predict marks in examination? The hypothesis was that they would not.
(iii) Does allocating higher proportions of marks to ongoing assessment increase pass rates? The hypothesis was that allocating higher proportions of marks to ongoing assessment was associated with higher marks and pass rates, and vice-versa.

### Objectives

(i) To compare the academic performance of students who passed the course in ongoing assessment and examinations.
(ii) To determine whether performance in ongoing assessment was a predictor of performance in examinations.
(iii) To consider how proportioning marks, between ongoing assessment and examinations, affected the overall marks and pass rates for the passing and failing students.

## Methods

This study was of nursing students in a pharmacology course. The study was repeated in a second semester and second year to determine whether the findings were consistent and ongoing.

### Study design

This is a descriptive study of the relationship between mark allocation to examinations and ongoing assessment (an assignment and/or tutorials) and the academic performance of nursing students in a pharmacology course.

### Research setting

The research was undertaken in an Australian university, where students are typically required to achieve an overall mark of 50% to pass a course and passing grades are 4 (overall mark, 50–64%), 5 (65–74%), 6 (75–84%) and 7 (≥ 85%). Thus, in the pharmacology course, students with < 50% of the overall marks failed the course.

### Course details

In the pharmacology course, 40% of the total marks were allocated to ongoing assessment, which had two components; tutorials and an assignment, both of which were allocated 20% of the marks. The tutorials were both formative and summative and were held weekly in classes of 25 students divided into groups of 5. Prior to the tutorials, the students were given a worksheet, which related to the lecture content for the week before the tutorial. Half of the tutorial marks were given for the completed worksheet, marked at the tutorial. The worksheet preparation was unsupervised and could be undertaken alone or in groups. The other half of the tutorial marks was a group mark for performance at the tutorial, which included questioning by the tutor of individuals and the group about the content of the student preparation.

The second 20% of the ongoing assessment was a summative written case-study assignment undertaken outside of class. This case study was an extension of the lecture content in medicines for cardiovascular disease and diabetes. It was a series of questions relating to a case of a person with complex cardiovascular and diabetic issues requiring short/essay answers.

The first part of the course was of principles of pharmacology and the second part was of systematic pharmacology. There were two examinations to make up the 60% for examinations. Firstly, there was a mid-semester examination covering the principles (20% multiple choice questions MCQs, 5% Short Answer Questions) and, secondly, there was a final examination of systematic pharmacology (35% MCQs).

The pharmacology course, taken by nursing students in the semesters of 2014 and 2015, had the same content and teacher. In both years, the pharmacology course enrolled ~ 250 students in semester 1 and ~ 360 students in semester 2, and some of these students withdrew or did not complete.

### Data collection procedures

The author was the coordinator of the pharmacology course, and as such had access to the Microsoft Excel sheets of the marks associated with the course. This data was starting point for the following analysis. In the analysis, *P* ≤ 0.05 were considered significant for both Student’s t-test and Odds ratios.

### Data analysis for objective 1: comparing academic performance in ongoing assessment and examinations

For the students, who successfully passed the course, average grades ± SEM were determined. The marks for the individual components ongoing assessment (combined and separated tutorials and assignment) and examinations were totalled, the total expressed as a percentage, and then the percentages were averaged. The percentages for individuals in examinations and ongoing assessment were compared by Students paired t-test, and the percentages for different cohorts in each component of the assessment were compared by Students unpaired t-test. Mean values were also determined.

### Data analysis for objective 2: regression line analysis for the passing students to determine whether performance in ongoing assessment was a predictor of performance in examinations

Regression line analysis was undertaken using Microsoft Excel. Thus, the marks for individual students in examinations were plotted against their marks in ongoing assessment (combined and separated tutorials and assignment). The equation for the regression line (y = ax + b), where ‘a’ is the slope of the line, and the R^2^ values were also given. In regression, the R^2^ coefficient of determination is a statistical measure of how well the regression line approximates the real data points, with an R^2^ of 1 indicating the regression line perfectly fits the data. To determine the strength of the association, the Pearson’s correlation (r) was determined and coefficients of 0–0.19 were considered very weak, 0.2–0.39 weak, 0.4–0.59 moderate, 0.6–0.79 strong, 0.8–1.0 very strong [21].

For all the students who completed the course (i.e. successful and failing students), modelling was undertaken to determine the effect of changing the marking proportions from 40% ongoing assessment/60% examinations had on the pass/failure rates and overall grades. The proportions modelled were changed to (i) 60% for ongoing assessment and 40% for examinations, (ii) 80% assessment /20% examination, (iii) 100% assessment /0% examination, (iv) 20% assessment/80% examination and (v) 0% assessment/100% examination. Mean values ± SEM were determined. Students who achieved less than 50% in the ongoing assessment or examinations were considered to have failed that component for both the actual and modelled data.

### Data analysis for objective 3: how proportioning marks, between ongoing assessment and examinations, affected the overall marks and pass rates for the passing and failing students

Students who achieved less than 50% in the ongoing assessment or examinations were considered to have failed that component; failure rates for each component were compared by Odds ratio using the online Odd ratio calculator; https://www.medcalc.org/calc/odds_ratio.php.

### Ethical consideration

Ethical approval was obtained for this project from the Human Research Ethics Committee (UHREC) at Queensland University of Technology; Ethics Approval Number 1900000541. The UHREC is constituted and operates in accordance the National Statement on Ethical Conduct in Human Research (2007) and registered by the National Health and Medical Research Council (Australia). Under this approval, consent from individual students was waived. Student anonymity was achieved by removing names and students’ IDs from the marks data prior to the study.

## Results

For completing students, the passing rate was > 90% and the failure rate was < 10% (Table 1).

**Table 1:** Actual and modelled data of overall marks, grades and passing/failing percentages

		2014				2015			
**Semester 1, Data type**	**% ongoing assessment/% exam**	**Overall mark (No of students)**	**Grade (No of students)**	**Additional students passing**^**a**^ **(% passing)**	**Additional students failing**^**b**^ **(% failing)**	**Overall mark (No of students)**	**Grade (No of students)**	**Additional students passing**^**a**^ **(% passing)**	**Additional students failing**^**b**^ **(% failing)**
Actual	40%/60%	66.5 ± 0.7 (215)	4.9 ± 0.1 (215)	(95%)	(5%)	66.3 ± 0.6 (197)	4.8 ± 0.1 (197)	(93%)	(7%)
Modelled	60%/40%	70.2 ± 0.7 (217)	5.1 ± 0.1 (217)	2/11 (96%)		69.2 ± 0.6 (200)	5.0 ± 0.1 (200)	3/15 (93%)	
Modelled	80%/20%	73.2 ± 0.7 (218)	5.4 ± 0.1 (218)	3/11 (97%)		71.6 ± 0.7 (209)	5.2 ± 0.1 (209)	9/15 (97%)	
Modelled	100%/0%	75.5 ± 1.0 (220)	5.8 ± 0.1 (220)	5/11 (97%)		74.6 ± 0.6 (213)	5.5 ± 0.1 (213)	13/15 (99%)	
Modelled	20%/80%	65.1 ± 0.7 (196)	4.7 ± 0.1 (196)		19/226 (8%)	65.3 ± 0.8 (191)	4.8 ± 0.1 (191)		16/215 (7%)
Modelled	0%/100%	64.0 ± 0.8 (171)	4.7 ± 0.1 (171)		44/226 (20%)	65.8 ± 0.8 (167)	4.8 ± 0.1 (167)		40/215 (19%)
**Semester 2, Data type**									
Actual	40%/60%	66.5 ± 0.7 (323)	4.8 ± 0.1 (323)	(92%)	(8%)	67.0 ± 0.5 (343)	4.8 ± 0.1 (343)	(94%)	(6%)
Modelled	60%/40%	70.8 ± 0.5 (332)	5.2 ± 0.1 (332)	9/27 (94%)		71.6 ± 0.6 (348)	5.2 ± 0.1 (348)	5/22 (95%)	
Modelled	80%/20%	74.9 ± 0.6 (337)	5.5 ± 0.1 (337)	14/27 (96%)		76.3 ± 0.6 (353)	5.7 ± 0.1 (353)	10/22 (97%)	
Modelled	100%/0%	77.9 ± 0.5 (335)	5.9 ± 0.1 (335)	12/27 (96%)		80.5 ± 0.6 (355)	6.0 ± 0.1 (355)	12/22 (97%)	
Modelled	20%/80%	65.0 ± 0.6 (296)	4.8 ± 0.1 (296)		27/350 (8%)	64.7 ± 0.6 (312)	4.7 ± 0.0 (312)		31/365 (9%)
Modelled	0%/100%	64.7 ± 0.7 (238)	4.8 ± 0.1 (238)		85/350 (25%)	63.8 ± 0.7 (259)	4.6 ± 0.1 (259)		84/365 (23%)

^a^Additional students passing of the completing students who had failed (% passing of completing students)

^b^Addition students failing of the completing students (% failing of completing students)

Mark values are the mean ± SEM (number of students)

### Comparison of marks for examinations and ongoing assessment for passing students

The average grade at ~ 4.8 (Table 1) and examination marks at 58–60% (Table 2) were similar between years and cohorts. There were only small variations between semesters for ongoing assessment (Table 2). Students in each cohort obtained significantly lower marks, ~ 15–20% point difference, in examinations than ongoing assessment (Table 2). Dividing the ongoing assessment showed that students obtained significantly lower marks, 9–19% point difference, in assignments than in tutorials (Table 2).

**Table 2:** For students who passed the course, percentage marks for examinations and ongoing assessment

Year and semester	Percentage Marks in each component (number of students)					
**2014**	Examinations	Ongoing assessment	Paired t-test	Tutorials	Assignment	Paired t-test
1	57.7 ± 0.9 (215)	72.4 ± 0.5 (215)	*P* < 0.0001	82.3 ± 1.1 (215)	71.2 ± 1.0 (215)	*P* < 0.0001
2	58.9 ± 0.7 (323)	77.9 ± 0.6 (323)	*P* < 0.0001	83.3 ± 0.7 (323)	74.2 ± 0.7 (323)	*P* < 0.0001
Unpaired t-test	Not significantly different	*P* < 0.0001		Not significant different	*P* < 0.0001	
**2015**						
1	60.1 ± 1.0 (207)	72.6 ± 0.9 (207)	*P* < 0.0001	82.3 ± 1.5 (207)	63.4 ± 1.5 (207)	*P* < 0.0001
2	58.5 ± 0.7 (343)	80.7 ± 0.6 (343)	*P* < 0.0001	83.4 ± 0.7 (343)	75.1 ± 0.7 (343)	*P* < 0.0001
Unpaired t-test	Not significantly different	*P* < 0.0001		Not significantly different	*P* < 0.0001	

Each value is the mean ± SEM (number of students)

Unpaired t-test is between semesters 1 and 2

Paired t-tests are between examinations and ongoing assessment marks, and between tutorial and assignment marks

Despite passing the pharmacology course overall by obtaining ≥50% of the total marks available, some of these students failed the individual components, by obtaining < 50%. Thus, the failure rates for the examinations ranged from 19 to 26% (Table 3). These examination failure rates for examinations were much higher than for the ongoing assessment; 0–1.6% (Table 3). None of the students who passed the pharmacology course failed the tutorial component. Thus, the failure rates in ongoing assessment were due to failure in the assignment component, which ranged from 3 to 6% (Table 3).

**Table 3:** For students who passed the course, failure rates in examinations and ongoing assessment

	Examinations	Ongoing assessment		Assignment
**2014**				
	Failure Rates (percentage)	Failure Rates (percentage)	Odds ratio/ *P* value	Failure Rates (percentage)
**Semester 1**	43/215 (20.0%)	3/215 (1.4%)	17.7/ *P* < 0.001	7/215 (3.3%)
**Semester 2**	85/323 (26.3%)	5/323 (1.6%)	21.4/ *P* < 0.001	8/323 (2.5%)
**2015**				
**Semester 1**	39/197 (19.8%)	0/197 (0%)	53.5/ *P* < 0.01	13/197 (6.6%)
**Semester 2**	86/343 (25.1%)	3/343 (0.9%)	37.9/*P* < 0.001	18/343 (5.2%)

Odds ratios are between examinations and ongoing assessment

Failure rates were number of student with less than 50%/total number of students who passed the unit (percentages)

### For the passing students, regression line analysis and Pearson’s correlation coefficients

**Fig. 1:**
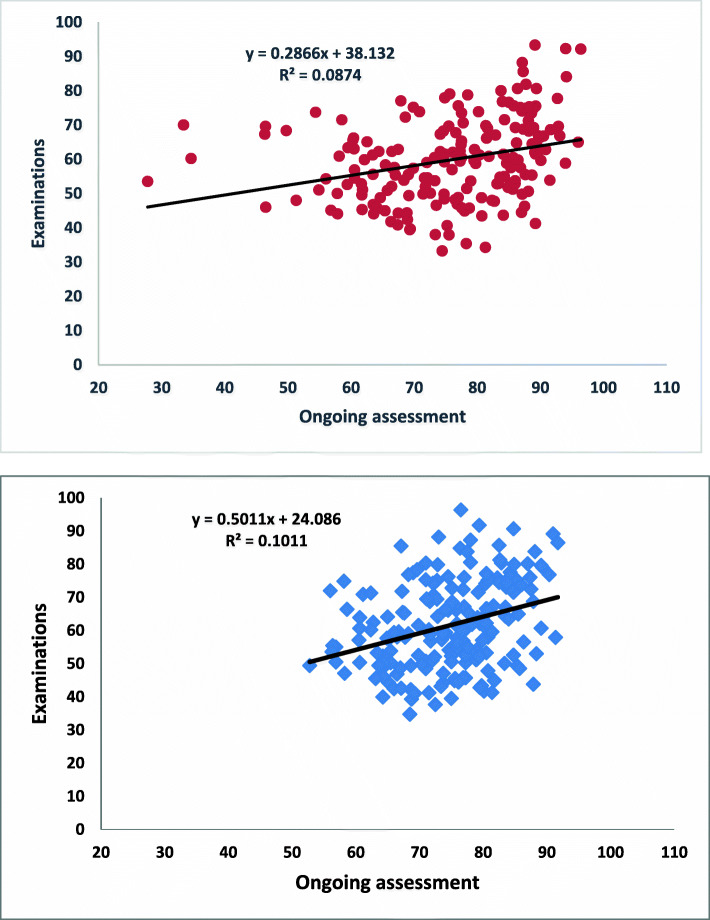
Regression analysis between the percentage marks in examinations and ongoing assessment in semester 1 of 2014 (top) and 2015 (bottom). The equation for the fitted line, and the R^2^ value are given on each graph

Regression line analysis was undertaken to determine whether performance in ongoing assessment was a good predictor of performance in the examinations. A good correlation would be indicated by the slopes of ~ 1 and R^2^ values would also be ~ 1. However, as students obtained significantly lower marks in examinations than in ongoing assessment (Table 2), it was predicted that there would be a poor fit of the data in regression line analysis, and this was the case (Fig. 1, Table 4).

**Table 4:** For students who passed the course, values from linear regression of examinations vs ongoing assessment

	2014				2015			
Semester	(n)	Exams vs ongoing assessment	Exams vs tutorial	Exams vs assignment	(n)	Exams vs ongoing assessment	Exams vs tutorial	Exams vs assignment
	**Slopes**							
1	215	0.287	0.569	0.322	197	0.501	0.065	−0.028
2	323	0.593	0.225	−0.001	343	0.303	0.134	0.244
	**R**^**2**^ **values**							
1	215	0.087	0.026	0.111	197	0.101	0.005	0.012
2	323	0.215	0.052	2E-06	343	0.062	0.019	0.064
	**Pearson’s correlation values**							
1	215	0.320	0.160	0.334	197	0.318	0.230	0.113
2	323	0.480	0.228	0.479	343	0.249	0.253	0.256

Values given are slopes, R^2^ values and Pearson’s correlation coefficients (n) = number of students who passed the course

Pearson’s correlation coefficients showed there was a weak correlation between the marks for examinations and ongoing assessment for three of the four semesters, and a moderate correlation for the other seminar (seminar 1 in 2014); Table 4. Dividing the ongoing assessment into tutorials or assignment marks also showed a poor line-fit to examination data to a line (Table 4). The correlations between tutorial and examination marks were weak, and between the assignment and examinations, very weak to moderate (Table 4).

### Modelling changing the proportional allocation of marks between ongoing assessment and examinations

The modelling changing the allocation of marks, from ongoing assessment to examinations and vice-versa, gave consistent results for all four cohorts of nursing students. Decreasing the allocation of marks to examinations increased the number of students who would have passed the course (Table 1). As the passing rates in the course were high (≥ 92%), there was little possibility of increasing these rates, and the modelling only resulted in a maximum of 2–6 percentage point increases (Table 1). Conversely, increasing the allocation of marks to examinations would have dramatically increased the number of students who failed the course (Table 1). The failure rates were low (≤ 8%) and were increased up to a maximum of 12–17 percentage points in the modelling (Table 1).

## Discussion

The three major findings of this study of nursing students in a pharmacology course are that for the passing students (i) marks are higher for ongoing assessment than examinations and (ii) there are very weak to moderate relationships between marks obtained in examination and ongoing assessment, and for completing students (iii) increasing the marks allocated to examinations decreased the number of students who passed the course, whereas decreasing the examination marks increased the number of students passing.

### Marks are higher for ongoing assessment than examinations

This is the first study to show that marks for ongoing assessment are higher than for examinations for nursing students in a pharmacology course. Similar findings have been made previously for bioscience courses being undertaken by nursing students [22] or science students [14] and confirms previous findings of higher marks for ongoing assessment at the program level [9–12].

There are several possible reasons for this disparity between marks in examinations and ongoing assessment. The most obvious of these is that the examination results represent those of the individual student, whereas the ongoing assessment marks may represent that of individuals or groups of students. In the present study, the tutorial mark of 20% is partly a group mark and is composed of 10% for unsupervised preparation/homework, which can be individual or group, and 10% for participation, which is a group mark. This makes it possible that the performance of weak students, and their marks in tutorials, to be artificially enhanced by better students in the group. The assignment component of the ongoing assessment (20%) should represent work undertaken by the individual student, but as this was unsupervised, there was nothing preventing students colluding. One way to overcome this would be to remove group work from courses. However, it is well known that group work is very important skill for nursing students. Thus, we need to be able to overcome this ongoing problem with assessing individuals in group work [23, 24] or use an alternative approach to make sure that students do not pass courses based on the work done by others in ongoing assessment.

For group assignments, self- and peer-rating has been used to overcome varying contributions by students in the humanities [25] and in postgraduate nursing/midwifery studies [26]. However, this method is not usually applied to weekly tutorials for students, including nursing students. When it was applied to problem-based learning tutorials for medical students, it was shown that self-ratings did not correlate, and peer- ratings only weakly correlated, with tutor-ratings of the students [27]. Thus, it is not proven that this method gives a reliable outcome of the student’s achievements in weekly tutorials. Furthermore, it would be very time consuming and expensive to undertake such assessment for weekly hourly tutorials in a large cohort. For instance, the pharmacology tutorials for nursing students in the present study were weekly over 13 weeks, in groups of 25, for cohorts of 250 or 350 students. Thus, self- and peer-ratings of tutorials are not routinely undertaken for large groups on a regular basis.

In the pharmacology course, 55% of the 60% of the marks allocated to examinations were in the form of MCQs. When MCQs are used, the fairest option is to focus on the number of questions attempted and penalize wrong answers, as with this option, blind guessing will on average not help the student [28]. Many universities, including the one that this study was undertaken at, do not deduct marks for incorrectly answered MCQs, and this inflates the MCQ marks [28]. In the pharmacology course studies, this could have inflated the marks for MCQs by ~ 20% and the overall mark in in the examination by 11% of the 60% of marks. Thus, the students who fail the examination in pharmacology by achieving less than 30% of the 60% of marks available are clearly demonstrating a poor knowledge of pharmacology, especially as the some of the marks may be due to blind guessing.

### Performance in ongoing assessment is a very weak to moderate predictor of performance in exams

In this study, we showed that for nursing students in pharmacology, marks in a written assignment were very weak to moderate predictors of performance in examinations. A previous study showed a weak correlation (like this study, using Pearson’s coefficient) between marks in a research project and the final examination in a pharmacy course [13]. It would be of interest to know whether this finding relating to assignments/projects applies to students in other disciplines.

In addition, the present study showed that marks in tutorials, which included a homework component, are not good predictors of academic performance in examinations. This is the first time that this has been shown for nursing students or in a pharmacology course. However, this finding is not consistent for all disciplines, as marked tutorials have been shown to improve marks for courses in calculus, macroeconomics [16], finance [17], and law [20].

### Altering the marks allocated to examinations changed the number of students who failed or passed

Increasing the marks allocated to examinations increased the number of students who failed the course and decreased the number who passed. With the allocation of marks of 60% to examinations and 40% to ongoing assessment, in the present study, the number of students who failed the pharmacology course was low (5–8%). With this low failure rate, the likelihood of increasing the passing rate by changing the allocation of marks was low, and our modelling confirmed this by showing that the passing rate could only be increased by 2–6 percentage points by increasing the marks allocated to ongoing assessment. With this allocation, the passing rate was high, 92–95%, and this occurred despite 20–26% of students failing the examination component of the course.

The major finding of the modelling part of our study was to show that increasing the marks allocated to examinations would have decreased the number of students who passed the course in pharmacology, with 19–25% failing overall if all the marks had been allocated to the examination. In Australia, the allocation of marks for examination in pharmacology or pharmacology-related courses from nursing programs ranges is variable (85%, University of Adelaide; 70%, University of Queensland; 50%, Edith Cowan University, RMIT University; 40% University of Tasmania [2–6]). Thus, if the standard trend of there being higher marks in ongoing assessment than examination occurs in these courses, for the same marks in ongoing assessment and examinations, a smaller percentage of students enrolled at Adelaide where examination marks predominate, would have been successful than if they had been enrolled at Tasmania, where marks for ongoing assessment predominates.

Although our modelling was done for a pharmacology course, the findings will apply to any course where the students have weaker outcomes in examinations than ongoing assessment, which is common [10–13]. As, to our knowledge, there are no previous studies of the either the relationship between marks in examination and ongoing assessment in an individual course, or of modelling the effect of changing the allocation of marks, for nursing or other students, these are novel findings.

### Implications of these results

As marks are higher for ongoing assessment than examinations, the concern is that the nursing students, who pass the ongoing assessment by obtaining 50% of the allocated marks, but not the examinations, may not have assimilated the necessary knowledge in pharmacology or other courses, to continue their program of study. Thus, the disparity between marks in examinations and ongoing assessment needs to be considered, and methods introduced to overcome this. One possible practical solution to this dilemma of whether students who pass ongoing assessment but fail examination, should be allowed to pass courses and progress in their studies, would be to make it compulsory for the students to pass the examination component of the course.

These findings have implications for those countries (Australia, UK, Republic of Ireland, New Zealand) where performance in undergraduate ongoing assessment is partly used to determine whether nursing students/graduates go on to clinical practice. In Australia, assessment for nursing students is commonly a mixture of ongoing assessment and examinations to give a Grade Point Average (GPA), and for many nursing courses/programs, most marks are from ongoing assessment. Thus, in the present nursing program at the university where the present study was undertaken there are 23 compulsory and one elective course. Seven of the courses are off-campus (practicums) and are marked as satisfactory or not satisfactory. Of the remaining 16 compulsory courses, 8 have no examinations, and 78% of marks are allocated to ongoing assessment and only 22% to examinations. It seems likely that the number of students who failed the examination components in our Australian university but passed the program overall, would have failed the NCLEX-RN examinations in USA system and not have been registered. Further consideration needs to be given as to whether students in Australia, who do not undertake or fail examinations, are fit to practice.

One possible practical solution to this dilemma of whether students who pass ongoing assessment but fail examination, should be allowed to pass courses and progress in their studies, would be to make it compulsory for the students to pass the examination component of the course. In addition, studies need to be undertaken that consider the relationship between success in undergraduate courses and clinical practice. Another practical solution is to adopt the system used in the USA, where after completion of an undergraduate course in nursing, success in a national examination, NCLEX-RN, is a requirement for clinical practice.

### Limitations

A limitation of this study is that it only used basic statistical analysis with Microsoft Excel, and more complex analysis could have been undertaken with other statistical packages (STATA, R, SPSS). However, the major limitation of this study is that it is of a single course in pharmacology, and that some of the findings may not relate to other courses being undertaken by nursing or non-nursing students. However, we have previously shown a similar reliance of marks in ongoing assessment for the overall success of nursing students in a bioscience course [22]. Also, the findings of the present study may apply to any course where students obtain significantly lower marks in examinations than ongoing assessment. However, for many courses, we do not know whether marks are lower for examinations than ongoing assessment for nursing or non-nursing students. Thus, similar analysis needs to be undertaken of other courses to determine whether the findings are specific to science courses for nursing students or can be related to other courses for nursing and non-nursing students.

## Conclusions

More attention needs to be given to the allocation of marks between ongoing assessment and examinations. Marks in ongoing assessment may be a poor indicator of success in examinations. Students can fail the examination component by obtaining 50% or less in the examination, but pass the course, and increasing the marks allocated to ongoing assessment accentuates this finding. Students, who pass the course but not the examinations, may not have assimilated the necessary knowledge to continue in their program. Additionally, some of the passing students may have passed overall due to work done by others in ongoing assessment. It is suggested that it should be compulsory for the students to pass the examination component of the course.

## Data Availability

The datasets used and/or analysed during the current study are available from the corresponding author on reasonable request.

## Abbreviations

NCLEX-RN: National council Licensure Examination-Registered Nurse
IDs: Student numbers
r values: Pearson’s correlation coefficients
SEM: Standard error of mean
MCQs: Multiple choice questions
